# Supplementary material for: Antenatal depression and its relationship with birth outcomes and postnatal depression in Rural India: A longitudinal study
Source: PLoS One. 2026 Mar 19;21(3):e0344176. doi: 10.1371/journal.pone.0344176 (PMC13001972; doi:10.1371/journal.pone.0344176)
Supplement: S1 File — S2 Table. Association of obstetric factors with AND. S3 Table. Association of spousal factors with AND. S4 Table. Identifying predictors of AND: logistic regression analysis. S5 Table. Association of Birth outcomes with AND. S6 Table. Association of birth outcomes with postnatal depression. (DOCX) [file pone.0344176.s001.docx]

| **Background Information** | **Antenatal depression** | | **P Value** |  | **Antenatal depression** | | **P Value** |
| --- | --- | --- | --- | --- | --- | --- | --- |
| **Respondent Age** | **No** | **Yes** | **0.912** | **Educational attainment** | **No** | **Yes** | **0.064** |
| <18 | 27.78 (5) | 72.22 (13) |  | Illiterate | 17.39 (8) | 82.61(38) |  |
| 19-21 | 20 (12) | 80 (48) |  | Primary | 8.82(3) | 91.18 (31) |  |
| 22-25 | 20.87 (24) | 79.13 (91) |  | Middle | 16.22 (6) | 83.78 (31) |  |
| 25+ | 20.75 (11) | 79.25 (42) |  | Secondary | 21.88 (14) | 78.13 (50) |  |
| **Age at Marriage** |  |  | **0.158** | Higher Secondary & Above | 32.31 (21) | 67.69 (44) |  |
| Below 18 | 19.18 (14) | 80.82 (59) |  | **Working status** |  |  | **0.832** |
| 18-21 | 20 (31) | 90 (124) |  | Housewife | 20.5 (41) | 79.5 (159) |  |
| Above 21 | 38.89 (7) | 61.11 (11) |  | Agriculture own land | 28.57 (2) | 71.43 (5) |  |
| **Age at 1st Birth** |  |  | **0.85** | Informal work | 23.08 (9) | 76.92 (30) |  |
| Below 18 | 11.11 (2) | 88.89 (16) |  | **Spouse education** |  |  | **0.013** |
| 18-21 | 14.68 (16) | 85.32 (93) |  | Illiterate | 6.98 (3) | 93.02 (40) |  |
| 22-25 | 19.05 (4) | 80.95 (17) |  | Primary | 12.82 (5) | 87.18 (34) |  |
| 25 & above | 25 (1) | 75 (3) |  | Middle | 18.75 (9) | 81.25 (39) |  |
| **Caste categories** |  |  | **0.001** | Secondary | 31.48 (17) | 68.52 (37) |  |
| SC & ST | 12.6 (16) | 87.4 (111) |  | Higher Secondary & Above | 29.03 (18) | 70.97 (44) |  |
| OBC | 27.45 (28) | 72.55 (74) |  | **Family type** |  |  | **0.003** |
| General | 47.06 (8) | 52.94 (9) |  | Single | 9.33 (7) | 90.67 (68) |  |
| **Religion** |  |  | **0.47** | Extended type | 26.32 (45) | 73.68 (126) |  |
| Hindu | 19.8 (39) | 80.2 (158) |  | **Mass media Exposure** |  |  | **0.002** |
| Muslim | 24.32 (9) | 75.68 (28) |  | Yes | 39.02 (16) | 60.98 (25) |  |
| Christian | 33.33 (4) | 66.67 (8) |  | No | 17.56 (36) | 82.44 (169) |  |
| **Total 43.50 56.50  (107) (139)**  **Note:** **P<0.05 ** P<0.01 ***P<0.005 # frequencies are given in parenthesis* | | | | | | | |

**Supplementary Table 1 (S1 Table):**

**Bivariate analysis of socio-demographic characteristics associated with antenatal**

**depression (AND)**

**Supplementary Table 2 (S2 Table): Association of obstetric factors with AND**

| **Obstetric factors** | **Antenatal depression** | | **P value** |
| --- | --- | --- | --- |
| **Gestational age** | **No** | **Yes** | **0.928** |
| First trimester | 19.72 (14) | 80.28 (57) |  |
| Second trimester | 21.88 (35) | 78.13 (125) |  |
| Third trimester | 20 (3) | 80 (12) |  |
| **Gravida** |  |  | **0.003** |
| Primigravida | 30.85 (29) | 69.15 (65) |  |
| Multigravida | 15.13 (23) | 84.87 (129) |  |
| **History of pregnancy loss** |  |  | **0.896** |
| Yes | 20.59 (14) | 79.41 (54) |  |
| No | 21.35 (38) | 78.65 (140) |  |
| **Note:** **P<0.05 ** P<0.01 ***P<0.005 # frequencies are given in parenthesis* | | | |

**Supplementary Table 3 (S3 Table): Association of spousal factors with AND**

| **Spousal factors** | **Antenatal depression** | | **P value** |
| --- | --- | --- | --- |
| **IPV violence** | No | Yes | **0.000** |
| Yes | 12.57 (23) | 87.43 (160) |  |
| No | 46.03 (29) | 53.97 (34) |  |
| **Smoking consumption (incl. stimulants)** |  |  | **0.000** |
| Yes | 9.62 (10) | 90.38 (94) |  |
| No | 29.58 (42) | 70.42 (100) |  |
| **Alcohol consumption** |  |  | **0.006** |
| Yes | 16.17 (27) | 83.83 (140) |  |
| No | 31.65 (25) | 68.35 (54) |  |
| **Tobacco consumption(chewing)** |  |  | **0.000** |
| Yes | 12.41 (18) | 87.59 (127) |  |
| No | 33.66 (34) | 66.34 (67) |  |
| **Note:** **P<0.05 ** P<0.01 ***P<0.005 # frequencies are given in parenthesis* | | | |

**Supplementary Table 4 (S4 Table): Identifying predictors of AND: logistic regression analysis**

| **Variables** | **Model 1** | **Model 2** | **Model 3** | **Model 4** | **Model 5** |
| --- | --- | --- | --- | --- | --- |
| **Maternal age** |  |  |  |  |  |
| <20® |  |  |  |  |  |
| 20-24 | 1.201 | 0.659 | 1.132 | 1.201 | 1.075 |
|  | [0.552,2.612] | [0.281,1.546] | [0.516,2.487] | [0.526,2.739] | [0.486,2.380] |
| 25+ | 1.092 | 0.475 | 1.000 | 1.156 | 0.986 |
|  | [0.460,2.596] | [0.177,1.278] | [0.414,2.417] | [0.461,2.898] | [0.408,2.381] |
| **Working status** |  |  |  |  |  |
| Housewife® |  |  |  |  |  |
| Agriculture om own land | 0.326 | 0.319 | 0.294 | 0.291 | 0.360 |
|  | [0.063,1.680] | [0.058,1.760] | [0.055,1.561] | [0.054,1.559] | [0.068,1.916] |
| Informal work | 0.942 | 0.768 | 0.912 | 0.990 | 0.891 |
|  | [0.437,2.030] | [0.342,1.723] | [0.421,1.975] | [0.435,2.249] | [0.412,1.931] |
| **Mass media exposure** |  |  |  |  |  |
| Yes® |  |  |  |  |  |
| No | 2.426^**^ | 2.944^***^ | 2.328^**^ | 1.795 | 2.046^*^ |
|  | [1.183,4.972] | [1.385,6.256] | [1.131,4.788] | [0.827,3.895] | [0.980,4.270] |
| **Religion** |  |  |  |  |  |
| Hindu® |  |  |  |  |  |
| Muslim | 0.413^**^ | 0.343^***^ | 0.406^**^ | 0.559 | 0.565 |
|  | [0.191,0.893] | [0.153,0.767] | [0.187,0.881] | [0.243,1.285] | [0.247,1.293] |
| Christian | 1.114 | 1.037 | 1.137 | 0.771 | 1.034 |
|  | [0.325,3.815] | [0.283,3.795] | [0.329,3.933] | [0.217,2.743] | [0.297,3.592] |
| **Family type** |  |  |  |  |  |
| Extended family® |  |  |  |  |  |
| Single | 2.764^***^ | 2.528^***^ | 2.710^***^ | 2.043^**^ | 2.560^***^ |
|  | [1.436,5.321] | [1.294,4.940] | [1.405,5.227] | [1.026,4.069] | [1.318,4.971] |
| **Gravida** |  |  |  |  |  |
| Primigravida® |  |  |  |  |  |
| Multigravida |  | 3.512^***^ |  |  |  |
|  |  | [1.826,6.754] |  |  |  |
| **History of pregnancy loss** |  |  |  |  |  |
| No**®** |  |  |  |  |  |
| Yes |  |  | 1.407 |  |  |
|  |  |  | [0.746,2.654] |  |  |
| **IPV during pregnancy** |  |  |  |  |  |
| No® |  |  |  |  |  |
| Yes |  |  |  | 5.797^***^ |  |
|  |  |  |  | [2.867,11.723] |  |
| **Alcohol consumption of spouse** |  |  |  |  |  |
| No® |  |  |  |  |  |
| Yes |  |  |  |  | 2.027^**^ |
|  |  |  |  |  | [1.077,3.817] |
| Exponentiated coefficients; 95% confidence intervals in brackets  ^*^ *p* < 0.10, ^**^ *p* < 0.05, ^***^ *p* < 0.01  *OR= Odds Ratio, CI= Confidence Interval, ®= Reference category* | | | | | |

**Supplementary Table 5 (S5 Table): Association of Birth outcomes with AND**

| **Birth outcomes** | **Antenatal depression** | | **P Value** |
| --- | --- | --- | --- |
| **Low birth weight** | No | Yes | **0.821** |
| Yes | 20.59 (7) | 79.41 (27) |  |
| No | 22.36 (36) | 77.64 (125) |  |
| **Premature Birth** |  |  | **0.896** |
| No | 22.48 (49) | 77.52 (169) |  |
| Yes | 20 (1) | 80 (4) |  |
| **Note:** **P<0.05 ** P<0.01 ***P<0.005 # frequencies are given in parenthesis* | | | |

**Supplementary Table 6 (S6 Table): Association of birth outcomes with postnatal depression**

| **Birth outcomes** | **Postnatal Depression** | | **P Value** |
| --- | --- | --- | --- |
| **Low birth weight** | **No** | **Yes** | **0.017** |
| No | 59.26 (96) | 40.74 (66) |  |
| Yes | 37.14 (13) | 62.86 (22) |  |
| **Type of delivery** |  |  | **0.082** |
| Vaginal delivery | 52.47 (85) | 47.53 (77) |  |
| C- section delivery | 68.57 (24) | 31.43 (11) |  |
| **Family reaction on childbirth** |  |  | **0.003** |
| Good | 59.88 (103) | 40.12 (69) |  |
| Normal | 27.27 (3) | 72.73 (8) |  |
| Bad | 21.43 (3) | 78.57 (11) |  |
| **Mothers of pressure a male child(s) wishing for a son but deliver daughter** |  |  | **0.001** |
| Yes | 30.43 (7) | 69.57 (16) |  |
| No | 91.67 (11) | 8.33 (1) |  |
| **Saved money for delivery** |  |  | **0.008** |
| Yes | 59.17 (100) | 40.83 (69) |  |
| No | 32.14 (9) | 67.86 (19) |  |
| **Birth experience** |  |  | **0.018** |
| Happy/good experience | 72.22 (39) | 27.78 (15) |  |
| Stressful | 50 (42) | 50 (42) |  |
| Traumatic/sad | 49.02 (25) | 50.98 (26) |  |
| **Gravida** |  |  | **0.027** |
| Primigravida | 65.33 (49) | 34.67 (26) |  |
| Multigravida | 49.18 (60) | 50.82 (62) |  |
| **ANC visits** |  |  | **0.044** |
| <4 | 46.99 (39) | 53.01 (44) |  |
| >4 | 61.4 (70) | 38.6 (44) |  |
| **Note:** **P<0.05 ** P<0.01 ***P<0.005 # frequencies are given in parenthesis* | | | |
