# Supplementary material for: Antenatal depression and its relationship with birth outcomes and postnatal depression in Rural India: A longitudinal study
Source: PLoS One. 2026 Mar 19;21(3):e0344176. doi: 10.1371/journal.pone.0344176 (PMC13001972; doi:10.1371/journal.pone.0344176)
Supplement: S2 File — (PDF) [file pone.0344176.s002.pdf]

Maternal Mental Health and Disrespect in Rural Jharkhand: An Exploratory Study of Chatra District

IDENTIFICATION

NAME OF THE RESPONDENT ______________________________

ADDRESS OF THE RESPONDENT____________________

____

SERIAL NUMBER

CONTACT NO. OF RESEARCHER: 91- 9812269537 GUIDE: 91-9769040275

RESPONDENT:

INTERVIEW DATE

Date		Month		Year			

RESULT STATUS OF THE INDIVIDUAL QUESTIONNAIRE

TOTAL PERSON IN HOUSEHOLD …...

TOTAL MALE…..

TOTAL FEMALE …..

INVESTIGATOR…...................................... FIELD EDITED BY…..

(For Women Only)

Informed Consent

Namaste, my name is___________(Name of the investigator) and I am a Ph.D. scholar at the International Institute for Population Sciences, Mumbai, India. As part of my PhD work, I will be collecting information from currently pregnant women about the types of maternal mental health issues, their associated factors, and outcomes. I would like to interview you and ask some questions for the purpose of the study. There are no right or wrong answers for these questions. All answers you provide in this survey will be recorded by me. No identifying information related to you will be provided to anyone. The survey data will be reported in a summarized manner only and will not reveal your identity to anyone else. It will be kept confidential and used for research purposes only. Your participation in this survey is voluntary. If I ask you a question you don't want to answer, just let me know, and I will go to the next question. Whenever you want to leave the interview, you are free to do so. This survey will take approximately 30-40 minutes to complete. May I proceed?

Thank you.

SIGNATURE OF THE INTERVIEWR______________________ DATE______________

1. CONSENT WITH SIGNATURE……………… ⟹ TO BE INTERVIEWED

2. CONSENT WITHOUT SIGNATURE………….. ⟹ TO BE INTERVIEWED WITHOUT SIGNATURE

3. REFUSAL………………………………… ⟹ END

Section1	Identification Information		
Q. No	Questions	Codes	Skip to
1	What day, month and year were you born? DD/MM/YYYY	Date of Birth____/____/_____<br>Don't know 98	
2	How old are you now? This would be age at last birthday.	Age in Years ______	
3	Have you ever attended school?	Yes .... 1<br>No..0 →	Q. No 5
4	What is the highest grade you completed?	Primary...................................1<br>Secondary................................2<br>Higher secondary.................... 3<br>Intermediate or 12th .... 4<br>Graduation.............................. 5<br>Post-graduation or more 6	
5	What is your religion?	Hindu .... 1<br>Muslim .... 2<br>Christian..................................3<br>Others __________________ 4<br>(Specify)	
6	What is the type of family?	Nuclear...................................1<br>Joint..2	
7	Have you been married once or more? If more, how many times,	Number of times	
8	How old were you at the time of your [first] marriage?	Age at first marriage ______<br>Don't know 98	
9	How old was your spouse at the time of marriage?	Age at marriage ______<br>Don't know 98	
10	What is/was the educational qualification of your spouse?	Illiterate ....1<br>Primary...................................2<br>Secondary................................3<br>Higher secondary.................... 4<br>Intermediate or 12th .... 5<br>Graduation.............................. 6<br>Post-graduation or more 7	

11	Whether you belong to a Scheduled Caste (SC), a Scheduled Tribe (ST), Other Backward Caste (OBC) or none of them?	Scheduled Caste .... 1<br>Scheduled Tribe..................... 2<br>OBC...................................... 3<br>Others ________________ 4<br>(Specify)	
12	Is your husband living with you now or is he staying elsewhere?	Living with me .... 1 →<br>Somewhere Else 2	Q. No. 15
13	What was the main reason for your spouse migration from the current place?	Economic ....1<br>Education................................2<br>Others __________________ 3<br>(Specify)	
14	How frequently he comes to meet you?	Every week.............................1<br>Every month .... 2<br>Once in a year.........................3<br>Others __________________ 9<br>(Specify)	
15	What is the main source of drinking water for members of your household?	Packaged Water.....................1<br>Own Piped water .... 2<br>Piped water Public..................3<br>Own well/Borewell................4<br>Well/Borewell Public .... 5<br>Surface sources......................6<br>Hand pump Public..................7<br>Own Hand pump .... 8<br>Others ________________ 96<br>(Specify)	
16	What kind of toilet facility do your household members generally use?	Open defecation..................... 1<br>Public Latrine .... 2<br>Septic tank/Flush System........ 3<br>Pit Latrine..............................4<br>Others ________________ 5<br>(Specify)	
17	What type of cooking fuel does your household mainly use?	Electricity .... 1<br>LPG/Natural Gas ....2<br>Biogas...................................3<br>Kerosene................................4<br>Coal/Lignite............................5<br>Charcoal .... 6<br>Wood .... 7<br>Straw/Shrubs/Grass................. 8<br>Agricultural. Crop waste......... 9	
18	What type of house is it?	*Kuchha* .... 1<br>Semi *Pucca*.............................2<br>*Pucca*.................................... 3	
19	Who owns this house?	Yourself................................ 1<br>Any usual member of the Household...............................2<br>Rented ....3<br>Others ________________ 4<br>(Specify)	

20	Does your household have:		Yes	No	
	Electricity?	Electricity?	1	2	
	A Mattress?	Mattress?	1	2	
	A Pressure cooker?	Pressure cooker?	1	2	
	A Chair?	Chair?	1	2	
	A Cot or Bed?	Cot or Bed?	1	2	

	A Table?	Table?	1	2	
	An Electric Fan?	Electric Fan?	1	2	
	A Radio or Transistor?	Radio or Transistor?	1	2	
	A Black and White Television?	Black and White Television?	1	2	
	A Colour Television?	Colour Television?	1	2	
	A Sewing Machine?	Sewing Machine?	1	2	
	A Mobile Phone?	Mobile Phone?	1	2	
	Any Landline Phone?	Landline Phone?	1	2	
	A Computer/laptop?	Computer?	1	2	
	Any Internet Facility?	Internet Facility?	1	2	
	A Refrigerator?	Refrigerator?	1	2	
	A Watch or wall/alarm clock?	Watch or wall/alarm clock?	1	2	
	A Bicycle?	Bicycle?	1	2	
	A Motorcycle or scooter?	Motorcycle or scooter?	1	2	
	An Animal-drawn cart?	Animal-drawn cart?	1	2	
	A Car/Jeep?	Car/Jeep?	1	2	
	A Water pump?	Water pump?	1	2	
	A Thresher?	Thresher?	1	2	
	A Tractor?	Tractor?	1	2	

21	Does anyone in this household own any agricultural land?	Yes....................................1<br>No.....................................2 →		Q. No 23
22	How much agricultural land does this household own?<br><br>(If not in acres specify size and unit)	Total (In acres)<br>Irrigated<br>Not Irrigated<br>Don't Know..........................98		
23	Does your household own any of the following animals?		Yes	No
	Cows, bulls, or buffaloes?	Cows/bulls/ buffaloes	1	2
	Goats?	Goats	1	2
	Sheep?	Sheep	1	2
	Chicken or ducks?	Chicken or ducks	1	2
	Pigs?	Pigs	1	2
24	Does this household have a Ration Card?	Yes, APL .... 1<br>Yes, BPL .... 2<br>Yes, *Antyodaya*.................... 3<br>No..4<br>Don't Know........................98		
25	What is/was the occupational status of your spouse?	No work/employment.........1 →<br>Own Agricultural work..........2<br>Agricultural/manual labour...3<br>Salaried Job........................... 4<br>Business................................5<br>Pension/rent/remittances........ 6<br>Any other (specify)............... 7		Q. No. 27
26	How much do your spouse earn in a month?	In Rupees..........................		
27	Have you done any work/job with salary/kind in the last 12 months?	Yes..1<br>No...2 →		Q. No. 32
28	How much do you earn in a month?	In Rupees..........................		
29	How much do your spouse earn in a month?	In<br>Rupees..........................		
30	Who decides how the money you earn will be used?	Mainly you........................... 1<br>Mainly your husband............. 2<br>You and your husband,<br>(jointly).................................3		
31	Would you say that the money that you earn:	More than your husband<br>earns .... 1<br>Less than what he earns .... 2<br>Same.....................................3		
32	Are you usually allowed to go to the market?	Alone.................................. 1<br>Only with someone else......... 2<br>Not at all...............................3		
33	Are you usually allowed to go to the health facility?	Alone.................................. 1<br>Only with someone else......... 2<br>Not at all...............................3		
34	Are you usually allowed to go to the places outside village/community?	Alone.................................. 1<br>Only with someone else......... 2<br>Not at all...............................3		

35	Do you have a bank or savings account that you yourself use?	Yes..1<br>No...2	
36	Are you currently using of these?	Newspaper.......................... A<br>Radio ....B<br>Television .... C<br>None of them .... D	
37	Do you have any mobile phone that you yourself use?	Yes..1<br>No...2	
38	Have you ever used the internet?	Yes..1<br>No...2	
39	Do you use smoking?	Yes.....................................1<br>No.......................................2 →	Q. No. 41
40	What is the frequency of smoking?	Everyday............................... 1<br>Once a week .... 2<br>Once a month .... 3<br>Occasionally 4	
41	Do you use alcohol?	Yes.....................................1<br>No.......................................2 →	Q. No. 43
42	What is the frequency of drinking?	Everyday................................ 1<br>Once a week .... 2<br>Once a month .... 3<br>Occasionally 4	
43	Do you consume gutkha/ Khani?	Yes.....................................1<br>No.......................................2 →	Q. No. 45
44	What is the frequency of this consumption?	Everyday................................ 1<br>Once a week .... 2<br>Once a month .... 3<br>Occasionally 4	
45	Does your spouse use any substance?	Alcohol.................................... A<br>Bidi/ cigarette/ Hukka................B<br>Gutkha/ khani .... C<br>None of the above.....................D →	Q. No. 50
46	What is the frequency of his smoking?	Everyday............................... 1<br>Once a week 2	

		Once a month .... 3<br>Occasionally 4	
47	What is the frequency of his drinking?	Everyday................................ 1<br>Once a week .... 2<br>Once a month .... 3<br>Occasionally 4	
48	Do your spouse consume gutkha/ Khani?	Yes...................................... 1<br>No..2 →	**Section 2**
49	What is the frequency of that consumption?	Everyday................................. 1<br>Once a week .... 2<br>Once a month .... 3<br>Occasionally 4	

Section 2	**Now, I would like to ask about all the pregnancies that you have had tillnow**		
50	How old were you at the time when your first child born?	Age in completed years	
51	How many live births you had tilldate?	Total<br>Male<br>Female	
52	Have you ever had a pregnancy that miscarried, was aborted, or ended in stillbirth?	Yes .... 1<br>No 2	
**Section 3**	**Now, I would like to ask about your last pregnancy/birth**		
53	How many months pregnant are you?	Months	
54	Did you register your last pregnancy?	Yes .... 1<br>No .... 2 →	Q. No 57
55	When was the pregnancy registered?	Within 12 weeks of pregnancy .... 1<br>After 12 weeks of pregnancy<br>…………………………………...2	
56	Pregnancy was registered withwhom?	Govt. Doctor .... 1<br>Private Doctor .... 2<br>ANM .... 3<br>*Anganwadi* Worker .... 4<br>ASHA .... 5<br>Other ____________________ 6	
57	Did you receive health check-ups or any treatment during this pregnancy?	Yes .... 1<br>No .... 2 →	Q. No 60
58	What is the distance to the nearest healthcare centre/medical facility?	Km……………………	

Where did you had the check-ups or treatment during pregnancy?

**Government**	Yes	No
Anganwadi/ICDS	1	2
Sub Health Centre	1	2
PHC	1	2
CHC	1	2
UHC/UHP/UFWC	1	2
Dispensary/Clinic	1	2
Hospital	1	2
Ayush Hospital/Clinic	1	2
Mobile Medical Unit	1	2
**Private**		
Dispensary/Clinic	1	2
Hospital	1	2
Nursing Home	1	2
Trust/Charity Hospital	1	2
**Public-Private**		
Sub Health Centre	1	2
PHC	1	2

<table>
<tr><td rowspan="11">59</td><td rowspan="11"></td><td>CHC</td><td>1</td><td>2</td><td rowspan="11"></td></tr>
<tr><td>UHC/UHP/UFWC</td><td>1</td><td>2</td></tr>
<tr><td>Dispensary/Clinic</td><td>1</td><td>2</td></tr>
<tr><td>Hospital</td><td>1</td><td>2</td></tr>
<tr><td>Ayush Hospital/Clinic</td><td>1</td><td>2</td></tr>
<tr><td>Mobile Medical Unit</td><td>1</td><td>2</td></tr>
<tr><td>Home</td><td></td><td></td></tr>
<tr><td>Own Home</td><td>1</td><td>2</td></tr>
<tr><td>Parent's Home</td><td>1</td><td>2</td></tr>
<tr><td>Other's Home</td><td>1</td><td>2</td></tr>
<tr><td>Others</td><td>1</td><td>2</td></tr>
<tr><td rowspan="8">60</td><td rowspan="8">Reasons for not having check-ups and treatment during pregnancy</td><td>Not necessary</td><td>1</td><td>2</td><td></td></tr>
<tr><td>Not customary</td><td>1</td><td>2</td><td></td></tr>
<tr><td>Cost too much</td><td>1</td><td>2</td><td></td></tr>
<tr><td>Too far/no transport</td><td>1</td><td>2</td><td></td></tr>
<tr><td>Non-dignified care</td><td>1</td><td>2</td><td></td></tr>
<tr><td>Abusive behaviour of health practitioners</td><td>1</td><td>2</td><td></td></tr>
<tr><td>Family did not allow</td><td>1</td><td>2</td><td></td></tr>
<tr><td>Lack of knowledge</td><td>1</td><td>2</td><td></td></tr>
<tr><td>61</td><td>Who accompanied you during the health check-ups?</td><td colspan="3">No one......................................1
Spouse......................................2
Father 3
Mother......................................4
Father-in-law 5
Mother-in-law............................ 6
Any other (Specify)................7</td><td></td></tr>
<tr><td>62</td><td>What is the
distance to the nearest healthcare centre/medical facility?</td><td colspan="3">Km…………</td><td></td></tr>
<tr><td rowspan="5">63</td><td rowspan="5">What was the cost of your antenatal care visit?</td><td>Doctor/OPD Fees</td><td colspan="2">In Rupees</td><td rowspan="5"></td></tr>
<tr><td>Medicine Fees</td><td colspan="2"></td></tr>
<tr><td>Transport Cost (If Any)</td><td colspan="2"></td></tr>
<tr><td>Hospital Charge</td><td colspan="2"></td></tr>
<tr><td>Inpatient Cost (If Any)</td><td colspan="2"></td></tr>
<tr><td>64</td><td>Is your family happy with your Pregnancy?</td><td colspan="3">Yes 1
No ....2</td><td></td></tr>
</table>

65	Do your family encourage you to take care of health?	Yes .... 1<br>No 2	
66	Do your family help in daily work?	Yes .... 1<br>No 2	
67	Do your family take care of daily medicine?	Yes .... 1<br>No 2	
68	Do your family help you in treatment?	Yes .... 1<br>No 2	
69	You receive any care and support from your husband in your previous pregnancy?	Yes .... 1<br>No .... 2<br>If yes please specify…………	
70	Did you face any violence during the previous pregnancy?	Yes .... 1<br>No .... 2 →	Q. No. 73
71	What types of violence?	Physical violence .... 1<br>Sexual violence ....2<br>Psychological violence......... 3<br>Verbal violence ....4<br>Other (Mention) 5	
72	Who were the perpetrators of violence?	Husband .... 1<br>Mother-in-law.........................2<br>Father-in-law ....3<br>Sister-in-law ....4<br>Parents.................................5<br>Relatives............................6<br>Neighbor .... 7<br>Community people.............8<br>Other (Mention) 9	
73	Are you receiving any supplementary nutrition from the anganwadi centre?	Yes .... 1<br>No 2	
74	Which kind of complications did you face for this pregnancy, till now?	Vaginal bleeding .... A<br>Abdominopelvic pain ....B<br>Vomiting .... C<br>High fever ....D<br>Vaginal Discharge....................E<br>Cramps .... F<br>Swelling of face, palm and feet…. G<br>Excessive fatigue/weakness……H	
		Weakness or no… ....I<br>movement of fetus<br>Abnormal position of fetus........... J<br>High Blood Pressure .... K<br>Jaundice ....L<br>Others (Specify) .... M	

Section 4	**Now, I would ask you about your relationship with your household or family members**				
75	Say or do something to humiliate you in front of others?	Yes ….1<br>No……2	Often<br>1	some-times<br>2	Not in the last 12 Month<br>3
76	Threaten to hurt or harm you or someone close to you?	Yes ….1<br>No……2	Often<br>1	some-times<br>2	Not in the last 12 Month<br>3
77	Insult you or make you feel bad about yourself?	Yes ….1<br>No……2	Often<br>1	some-times<br>2	Not in the last 12 Month<br>3
78	Push you, shake you, or throw something at you?	Yes ….1<br>No……2	Often<br>1	some-times<br>2	Not in the last 12 Month<br>3
79	Twist your arm or pull your hair?	Yes ….1<br>No……2	Often<br>1	some-times<br>2	Not in the last 12 Month<br>3
80	Slap you?	Yes ….1<br>No……2	Often<br>1	some-times<br>2	Not in the last 12 Month<br>3
81	Punch you with his fist or with something that could hurt you?	Yes ….1<br>No……2	Often<br>1	some-times<br>2	Not in the last 12 Month<br>3
82	Kick you, drag you or beat you up?	Yes ….1<br>No……2	Often<br>1	some-times<br>2	Not in the last 12 Month<br>3
83	Try to choke you or burn you on purpose?	Yes ….1<br>No……2	Often<br>1	some-times<br>2	Not in the last 12 Month<br>3
84	Threaten or attack you with a knife, gun, or any other weapon?	Yes ….1<br>No……2	Often<br>1	some-times<br>2	Not in the last 12 Month<br>3
85	Has anyone from your family ever hit, slapped, kicked, or done anything else to hurt you physically while you were pregnant?	Yes.....................................1<br>No.....................................2			

**Section 5**	**Now I would like to ask about your mental health during this pregnancy**				
**Symptoms**	**No (1)**	**Sometimes (2)**	**Regularly (3)**	**Often (4)**	**Very often (5)**
**During the past week, did you suffer from:** **(Somatization)**					
**1.** Dizziness or feeling light-headed?					
**2.** Painful muscles?					
**3.** Fainting?					
**4.** Neck pain?					
**5.** Back pain?					
**6.** Excessive sweating?					
**7.** Palpitations?					
8. Headache?					
9. A bloated feeling in the abdomen?					
10. Blurred vision or spots in front of your eyes?					
11. Shortness of breath?					
12. Nausea or an upset stomach?					
13. Pain in the abdomen or stomach area?					
14. Tingling in the fingers?					

15. Pressure or a tight feeling in the chest?					
16. Pain in the chest?					
Distress<br>***17.*** Feeling down or depressed?					
18. Worry?					
19. Disturbed sleep?					
20. Lack of energy?					
(Anxiety)<br>***21.*** Sudden fright for no reason?					
22. A vague feeling of fear?					
23. Trembling when with other people?					
24. Anxiety or panic attacks?					
**During the past week, did you feel:**					
(Distress)<br>***25.*** Tense?					
26. Easily irritated?					
27. That you just can't do anything anymore?					
28. That you can no longer take any interest in the People and things around you?					
29. That you can't cope anymore?					

30. That you can't face it anymore?					
(Anxiety)<br>***31.*** Frightened?					
(Depression)<br>***32.*** That everything is meaningless?					
33. That life is not worthwhile?					
34. That you would be better off if you were dead?					
35. That you can't enjoy anything anymore?					
36. That there is no escape from your situation?					
**During the past week, did you:**					
(Distress)<br>***37.*** No longer feel like doing anything?					
38. Have difficulty in thinking clearly?					
39. Have difficulty in getting to sleep?					
(Anxiety)<br>***40.*** Have any fear of going out of the house alone?					
**During the past week:**					
(Distress)<br>***41.*** Did you easily become emotional?					
42. Did you ever fleeting images of any upsetting events that you have experienced?					
43. Did you ever have to do your best to put aside thoughts about any upsetting events?					

(Anxiety)<br>***44.*** Were you afraid of anything when there was<br>Really no need for you to be afraid?<br>(for instance, animal, heights, small rooms)					
45. Were you afraid to travel on buses, streetcars/<br>trams, subways or trains?					
46. Were you afraid of becoming embarrassed when with other people?					
47. Did you ever feel as if you were being threatened by unknown danger?					
48. Did you have to avoid certain places because they frightened?					
49. Did you have to repeat some actions a number of times before you could do something else?					
(Depression)<br>***50.*** Did you ever think "I wish I was dead"?					
**51.** Past history of any mental issue?	Yes .... 1 →<br>No.....................................2				Q. No 52
**52.** Have you diagnose or taken medicine for that for mental illness?	Yes .... 1<br>No.....................................2				
