# Supplementary material for: Antenatal depression and its relationship with birth outcomes and postnatal depression in Rural India: A longitudinal study
Source: PLoS One. 2026 Mar 19;21(3):e0344176. doi: 10.1371/journal.pone.0344176 (PMC13001972; doi:10.1371/journal.pone.0344176)
Supplement: S3 File — (PDF) [file pone.0344176.s003.pdf]

Maternal Mental Health and Disrespect in Rural Jharkhand: An Exploratory Study of Chatra District

IDENTIFICATION

NAME OF THE RESPONDENT ____

ADDRESS OF THE RESPONDENT______________________________

____

SERIAL NUMBER

CONTACT NO. OF RESEARCHER: 91- 9812269537 GUIDE: 91-9769040275

RESPONDENT:

INTERVIEW DATE

Date		Month		Year			

RESULT STATUS OF THE INDIVIDUAL QUESTIONNAIRE

TOTAL PERSON IN HOUSEHOLD …..

TOTAL MALE…..

TOTAL FEMALE …..

INVESTIGATOR….................................... FIELD EDITED BY…...

(For Women Only)

Informed Consent

Namaste, my name is___________ (Name of the investigator) and I am a Ph.D. scholar at the International Institute for Population Sciences, Mumbai, India. As part of my PhD work, I will be collecting information from currently pregnant women about the types of maternal mental health issues, their associated factors, and outcomes. I would like to interview you and ask some questions for the purpose of the study. There are no right or wrong answers for these questions. All answers you provide in this survey will be recorded by me. No identifying information related to you will be provided to anyone. The survey data will be reported in a summarized manner only and will not reveal your identity to anyone else. It will be kept confidential and used solely for research purposes. Your participation in this survey is voluntary. If I ask you a question you don't want to answer, just let me know, and I will go to the next question. Whenever you want to leave the interview, you are free to do so. This survey will take approximately 30-40 minutes to complete. May I proceed?

Thank you

SIGNATURE OF THE INTERVIEWR_____________________ DATE_____________

1. CONSENT WITH SIGNATURE……………… ⟹ TO BE INTERVIEWED

2. CONSENT WITHOUT SIGNATURE………….. ⟹ TO BE INTERVIEWED WITHOUT SIGNATURE

3. REFUSAL………………………………… ⟹ END

Postnatal Phase

Q. No	Questions	Codes			Remarks
1	How many antenatal visits you complete before the delivery?	No Visit .... 1<br>1……………………………2<br>2……………………………3<br>3……………………………4<br>4 or more................................. 5<br>Don't know….........................98			
2	Sex of the recent birth	Male...1<br>Female .... 2<br>Others 3			
3	Did you receive delivery care?	Yes.................................1<br>No .... 2 →			Q.No 6
4.	How the last birth took place?	Normal................................ 1<br>caesarean...............................2<br>Forceps/Assisted........................3			
5.	Where did you receive delivery Care?	Government Hospital…............... 1<br>Private Hospital .... 2<br>Home .... 3<br>Other..4<br>(specify)			
6	Desire for companion during labor and delivery?	Husband.................................1<br>Mother… .... 2<br>Father.....................................3<br>Mother-in- law........................4<br>Father-in-law.......................... 5<br>Others 78			
6	Reasons for not received delivery care?	Not necessary	1	2	
		Not customary	1	2	
		Cost too much	1	2	
		Too far/no transport	1	2	
		Non-dignified care	1	2	
		Abusive behaviour of health practitioners	1	2	
		Family did not allow	1	2	
		Lack of knowledge	1	2	
		Other	1	2	
7	Did you or your newborn receive a health check-up within two days after the delivery (post-natal care)?	Yes.................................1<br>No .... 2 →			Q. No 9
8	Where did you receive Postnatal Care?	Government Hospital…............... 1<br>Private Hospital .... 2<br>Home .... 3<br>Other..4<br>(specify)			
9	Reasons for not received Postnatal care?	Not necessary	1	2	
		Not customary	1	2	
		Cost too much	1	2	

		Too far/no transport	1	2	
		Non-dignified care	1	2	
		Abusive behaviour of health practitioners	1	2	
		Family did not allow	1	2	
		Lack of knowledge	1	2	
		Other	1	2	
10	Weight of child at the time of birth?	Yes...1<br>No..2 →			Q. 13
11	Weight in Kilogram?	Less than 1 kg…....................1<br>1 to 2 kg…..............................2<br>2 kg and above........................3			
12	During delivery, did you experience any of them?	Breech presentation .... 1<br>Prolonged labour .... 2<br>Excessive bleeding .... 3<br>None of them 4			
**Section 2: Now I would like to ask you about your birth preparedness**					
13	Did you receive any counselling about birth preparation from a health worker?	Yes...1<br>No.. 2			
14	Have you specified a delivery location?	Yes...1<br>No..2 →			
15	Which location did you specify for delivery?	Institutional delivery................ 1<br>Home Delivery… .... 2 →			Q. No.19
16	Who advised to go for institutional delivery?	ASHA worker.................1<br>Doctor.............................2<br>Family…..........................3<br>Other...............................4<br>(Specify)			
17	When you got pregnant, did you want to get pregnant at that time?	Yes.................................1<br>No................................. 2			
18	Did you had a pressure to have a male child?	Yes.................................1<br>No................................. 2			
19	Did you wanted last child Son and delivered daughter?	Yes.................................1<br>No................................. 2			
20	Did you save money for the delivery?	Yes...1<br>No.. 2			
21	Did you arrange transport facility to reach the hospital?	Yes...1<br>No.. 2			
22	Did you contact any skilled health worker?	Yes...1<br>No.. 2			

23	Have you approached anyone for blood donation?	Yes......1<br>No...... 2			
24	Did you buy the protection kit?	Yes......1<br>No...... 2			
25	Did you arrange food?	Yes......1<br>No...... 2			
26	Did you arrange the clothes?	Yes......1<br>No...... 2			
27	Do you know about the complications of childbirth?	Yes......1<br>No...... 2			
28	Have you had any obstetric complications?	Yes......1<br>No...... 2			
**Section 3: Now, the questions will be on your nutrition, delivery and postnatal care costs and incentives**					
29	What was the delivery cost?	A. Doctor/OPD Fees	In Rupees		
		B. Medicine Fees			
		C. Transport Cost (If Any)			
		D. Hospital Charge			
		E. Inpatient Cost (If Any)			
30	Are you the beneficiary of JSY scheme?	Yes......1<br>No...... 2 →			Q. No. 31
31	How much amount do you receive?	In Rupees……….			
32	Are you the beneficiary of Matru Vandana Yojna?	Yes......1<br>No...... 2 →			Q. No. 33
33	How much amount do you receive?	In Rupees……….			
34	How was the behavior of the medical centre/hospital (health care facility) staff during your delivery?	A. It was good:	Yes	No	
		B. Were talking out loud?	Yes	No	
		C. Were abusing?	Yes	No	
		D. were hitting?	Yes	No	
35	Did it happen that you were not able to eat the food you wanted?	Yes...... 1<br>No......2 →			Q. No. 39
36	How often were you unable to eat food items you wished to because of?	A. Accessibility	Yes	No	
		B. Availability	Yes	No	
		C. Affordability	Yes	No	
37	How often did you have to forego certain quantities of food intake because of?	A. Accessibility	Yes	No	
		B. Availability	Yes	No	
		C. Affordability	Yes	No	
38		A. Accessibility	Yes	No	
		B. Availability	Yes	No	

	How often did you have to skip your meals totally because of?	C. Affordability	Yes	No	
39	What was the postnatal cost?	A. Doctor/OPD Fees	In Rupees		
		B. Medicine Fees			
		C. Transport Cost (If Any)			
		D. Hospital Charge			
		E. Inpatient Cost (If Any)			

Section 4: Now I would like to ask you about your mental health

Symptoms	No (1)	Sometimes (2)	Regularly (3)	Often (4)	Very often (5)
**During the past week, did you suffer from:** **(Somatization)**					
**1.** Dizziness or feeling light-headed?					
**2.** Painful muscles?					
**3.** Fainting?					
**4.** Neck pain?					
**5.** Back pain?					
**6.** Excessive sweating?					
**7.** Palpitations?					
8. Headache?					
9. A bloated feeling in the abdomen?					
10. Blurred vision or spots in front of your eyes?					
11. Shortness of breath?					
12. Nausea or an upset stomach?					
13. Pain in the abdomen or stomach area?					
14. Tingling in the fingers?					

15. Pressure or a tight feeling in the chest?					
16. Pain in the chest?					
Distress<br>***17.*** Feeling down or depressed?					
18. Worry?					
19. Disturbed sleep?					
20. Lack of energy?					
(Anxiety)<br>***21.*** Sudden fright for no reason?					
22. A vague feeling of fear?					
23. Trembling when with other people?					
24. Anxiety or panic attacks?					
**During the past week, did you feel:**					
(Distress)<br>***25.*** Tense?					
26. Easily irritated?					
27. That you just can't do anything anymore?					
28. That you can no longer take any interest in the<br>People and things around you?					

29. That you can't cope anymore?					
30. That you can't face it anymore?					
(Anxiety) ***31.*** Frightened?					
(Depression) ***32.*** That everything is meaningless?					
33. That life is not worthwhile?					
34. That you would be better off if you were dead?					
35. That you can't enjoy anything anymore?					
36. That there is no escape from your situation?					
**During the past week, did you:**					
(Distress) ***37.*** No longer feel like doing anything?					
38. Have difficulty in thinking clearly?					
39. Have difficulty in getting to sleep?					
(Anxiety) ***40.*** Have any fear of going out of the house alone?					
**During the past week:**					
(Distress) ***41.*** Did you easily become emotional?					
42. Did you ever fleeting images of any upsetting events that you have experienced?					

43. Did you ever have to do your best to put aside thoughts about any upsetting events?					
(Anxiety)<br>***44.*** Were you afraid of anything when there was<br>Really no need for you to be afraid?<br>(for instance, animal, heights, small rooms)					
45. Were you afraid to travel on buses, streetcars/<br>trams, subways or trains?					
46. Were you afraid of becoming embarrassed when with other people?					
47. Did you ever feel as if you were being threatened by unknown danger?					
48. Did you have to avoid certain places because they frightened?					
49. Did you have to repeat some actions a<br>number of times before you could do<br>something else?					
(Depression)<br>***50.*** Did you ever think "i wish i was dead"?					
**51.** Past history of any mental issue?	Yes .... 1 →<br>No 2				**Q. 52**
**52.** Have you diagnose or taken medicine for that for mental illness?	Yes .... 1<br>No 2				
