# Supplementary material for: Antenatal depression and its relationship with birth outcomes and postnatal depression in Rural India: A longitudinal study
Source: PLoS One. 2026 Mar 19;21(3):e0344176. doi: 10.1371/journal.pone.0344176 (PMC13001972; doi:10.1371/journal.pone.0344176)
Supplement: S4 File — (DOCX) [file pone.0344176.s004.docx]

Inclusivity in global research

PLOS’ policy on inclusivity in global research aims to improve transparency in the reporting of research performed outside of researchers’ own country or community and ensures that PLOS publications reporting global research adhere to high standards for research ethics and authorship. Authors of relevant research articles may be asked to complete the questionnaire below, which outlines ethical, cultural, and scientific considerations specific to inclusivity in global research. This questionnaire may be requested when researchers have travelled to a different country to conduct research, if research uses samples collected in another country, research with Indigenous populations or their lands, or if research is on cultural artefacts. Researchers travelling to another country solely to use laboratory equipment will not normally be required to complete the questionnaire. However, the questionnaire can be requested at the journal’s discretion for any submission – if you have been requested to complete this questionnaire by the PLOS journal you submitted to, please do so.

Please complete the questionnaire below and include this as a Supporting Information file with your manuscript. Note that if your paper is accepted for publication, this checklist will be published with your article in the supporting information files. Please ensure that you reference the checklist in the main body of your manuscript. We suggest adding a subsection ‘Inclusivity in global research’ to your Methods section and adding the following sentence: “Additional information regarding the ethical, cultural, and scientific considerations specific to inclusivity in global research is included in the Supporting Information (SX Checklist)”

The questions have been designed to be applicable to a wide range of study types, and there are subsections for both human subjects research and non-human subjects research. If any of the questions are not relevant to your research please mark them as “N/A” as appropriate.

**Ethical considerations, permits and authorship**

*This section is applicable to all research types.*

Provide details as to who granted permissions and/or consent for the study to take place in the Methods section of your manuscript. This should include the names of **all** ethics boards, governmental organizations, community leaders or other bodies that provided approval for the study. If individuals provided approval refer to these people by their role or title but do not list their name(s).

Reported on page number: 7

The Institutional Review Board of the International Institute for Population Sciences (Student Research Ethics committee), Mumbai, India (approval number: IIPS/ACAD/SREC/D/IO-25/2023) granted the study's ethical approval. All participants provided written informed consent prior to data collection. The research followed institutional guidelines for ethical conduct. No additional permits or governmental/community leader approvals were required beyond institutional review.

If there were any deviations from the study protocol after approval was obtained please provide details of these changes in the Methods section of your manuscript.

There were no deviations from the original study protocol after obtaining ethical approval. All data collection and procedures were conducted as planned.

Did this study involve local collaborators that are residents of the country where the research was conducted or members of the community studied? If you do not have any authors from said communities, please provide

An explanation for this is below.

Yes, the study team included local collaborators. The first and second authors, who are based in India and affiliated with IIPS, Mumbai, handled the study design, fieldwork, data collection, manuscript writing, and supervision. Their involvement helped ensure the research was relevant to the local context and that data collection met community needs.

Everyone listed as an author should meet PLOS’ criteria for authorship and all individuals who meet these criteria should be included in the author byline, rather than the acknowledgements. For further information please see the journal’s Authorship Policy.

All individuals listed as authors meet PLOS’ criteria for authorship, contributing substantially to the conception, design, data collection, analysis, and/or drafting and revising the manuscript. No eligible contributors have been excluded from the author byline; all who meet the journal’s authorship requirements are appropriately listed as authors.

**Human subjects research (e.g. health research, medical research, cross-cultural psychology)**

Did you obtain written informed consent from a representative of the local community or region before the research took place? How did you establish who speaks for the community? Details of written informed consent obtained from study participants should be reported separately in the Methods section of your manuscript.

Yes, written informed consent was obtained from all individual study participants before data collection. Details of this consent process are reported in the methods section of the manuscript. Formal community-level consent was not separately obtained; instead, the research was facilitated by local Accredited Social Health Activists (ASHA workers) and health facility staff, who helped in verifying participant’s eligibility and supported community engagement. The study was also approved by the institutional ethics board (Student Research Ethics committee), which reviewed and approved all protocal for participant consent.

How did members of the local community provide input on the aims of the research investigation, its methodology, and its anticipated outcome(s)?

Community members were involved in the study through discussions with Accredited Social Health Activists (ASHA workers) and local health staff. These health workers helped explain the study’s purpose and questions in ways that fit local culture, which improved the study tools. A pilot survey with pregnant women in the community also provided useful feedback to adjust the methods and make the questionnaires clearer and more relevant. Overall, input from ASHAs, health staff, and local women was used to shape the study design, methods, and instruments so they matched local needs and practices.

When engaging with the local community, how did you ensure that the informed consent documents and other materials could be understood by local stakeholders?

To make sure local people clearly understood the consent forms and research materials, everything was translated into Hindi and explained in simple local language. Trained research assistants and ASHA workers were available to clarify doubts and answer questions in the local dialect. A pilot test with local women was also done to check the clarity of the materials and improve them before starting the main study.

Will the findings of the research be made available in an understandable format to stakeholders in the community where the study was conducted (e.g. via a presentation, summary report, copies of publications, etc.)? Please provide details of how this will be achieved.

Yes, the findings will be shared in easy-to-understand formats with local stakeholders. After completing the PhD, a summary report and key results will be shared with health staff, ASHA workers, and community members through meetings or presentations. Summaries will be translated into Hindi and explained in simple terms so that everyone can understand and use the results for iproving the existing health care ficilities. Copies of publications and brief reports will also be provided to community stakeholders and local health centers for wider sharing.

**Non-human subjects research using specimens/ animals collected as part of the study, or those housed in archival collections. Examples include archaeology, paleontology, botany and zoology.**

Did the permission you obtained from a local authority to perform the study include an agreement on access to outputs and benefit sharing? This may include procedures to enable fair distribution of the benefits and resources arising from the research performed. Please include any details of Prior Informed Consent and Benefit Sharing Agreements obtained. These may be required by field-specific regulations, for example the Convention on Biological Diversity (CBD) and the associated Nagoya Protocol.

N/A

If the material used in your study was imported, please A) provide the year it was imported and B) indicate whether permits were obtained to import/export the materials used, C) provide details of any permits obtained. If this information is not available, please indicate this.

N/A

If you used archival specimens, please state how the material used in your study was acquired by the institute it is held in and provide details of any permits obtained for the original excavations/ sample collection. If this information is not available, please indicate this.

N/A

How was the potential cultural significance of the materials collected in your study to local communities considered in your research design? Were Indigenous peoples and/or local researchers and institutions involved with archaeological excavations / collection of specimens? If so, please provide a description of their involvement.

N/A

If your manuscript includes photographs of human remains please indicate whether authors obtained permission from descendants or affiliated cultural communities to do so.

N/A
