# Supplementary material for: Antenatal depression and its relationship with birth outcomes and postnatal depression in Rural India: A longitudinal study
Source: PLoS One. 2026 Mar 19;21(3):e0344176. doi: 10.1371/journal.pone.0344176 (PMC13001972; doi:10.1371/journal.pone.0344176)
Supplement: Attachment — Submitted filename: Reviewer Comments Antenatal Depression.docx [file pone.0344176.s005.docx]

The reviewer commends the study on antenatal depression (AND), birth outcomes, and postnatal depression (PND) in rural India for its valuable insights, longitudinal design, and focus on an underserved population. Key recommendations include clarifying research hypotheses, addressing participant attrition, discussing statistical power limitations, and justifying the depression scale cutoff.

The reviewer suggests elaborating on the lack of significant findings between AND and birth outcomes, expanding discussion on cultural context and interviewer bias, and including comparative global literature.

Minor improvements involve refining the title, mentioning tool validation in the abstract, enhancing table clarity, and acknowledging additional limitations such as recall bias. Overall, minor revisions are recommended to improve clarity, contextualization, and policy relevance.
